# Temperature and Monsoon Tango in a Tropical Stalagmite: Last Glacial-Interglacial Climate Dynamics

**DOI:** 10.1038/s41598-018-23606-w

**Published:** 2018-03-29

**Authors:** Carme Huguet, Joyanto Routh, Susanne Fietz, Mahjoor Ahmad Lone, M. S. Kalpana, Prosenjit Ghosh, Augusto Mangini, Vikash Kumar, Ravi Rangarajan

**Affiliations:** 1 Departamento de Geociencias, Universidad de los Andes, Bogotá, Colombia; 2 Department of Thematic Studies, Environmental Change, Linköping University, 58183 Linköping, Sweden; 3 Department of Earth Sciences, Stellenbosch University, 7602 Stellenbosch, South Africa; 4 High-Precision Mass Spectrometry and Environment Change Laboratory, Department of Geosciences, National Taiwan University, Taipei, 10617 Taiwan; 5 CSIR-National Geophysical Research Institute, Hyderabad, 500007 India; 6 Centre for Earth Sciences, Indian Institute of Sciences, Bangalore, 560012 India; 7 Institut für Umweltphysik, INF 229, Heidelberg, 69120 Germany; 8 National Centre for Antarctic & Ocean Research, Goa, 403804 India

## Abstract

High-resolution paleoclimate data on stable isotopes in a stalagmite were coupled to glycerol dialkyl glycerol tetraethers (GDGTs). The Indian Summer Monsoon (ISM) transitioned from limited rainfall during the Last Glacial Maximum (LGM) to intense precipitation during early Holocene (22 to 6 ka). This was associated with changes in stalagmite growth, abundance of branched (br) and isoprenoid (iso) GDGTs, as well as δ^18^O, δ^13^C, Sr/Ca and GDGT-derived signals providing both temperature and moisture information. The reconstructed mean annual air temperature (MAAT) of the most modern stalagmite sample at ~19 °C, matches the surface and cave MAAT, but was ~4 °C lower during LGM. Warming at the end of LGM occurred before ISM strengthened and indicate 6 ka lag consistent with sea surface temperature records. The isotope records during the Younger Dryas show rapid progressions to dry conditions and weak monsoons, but these shifts are not coupled to TEX_86_. Moreover, change to wetter and stronger ISM, along with warmer Holocene conditions are not continuous indicating a decoupling of local temperatures from ISM.

## Introduction

**Figure 1:**
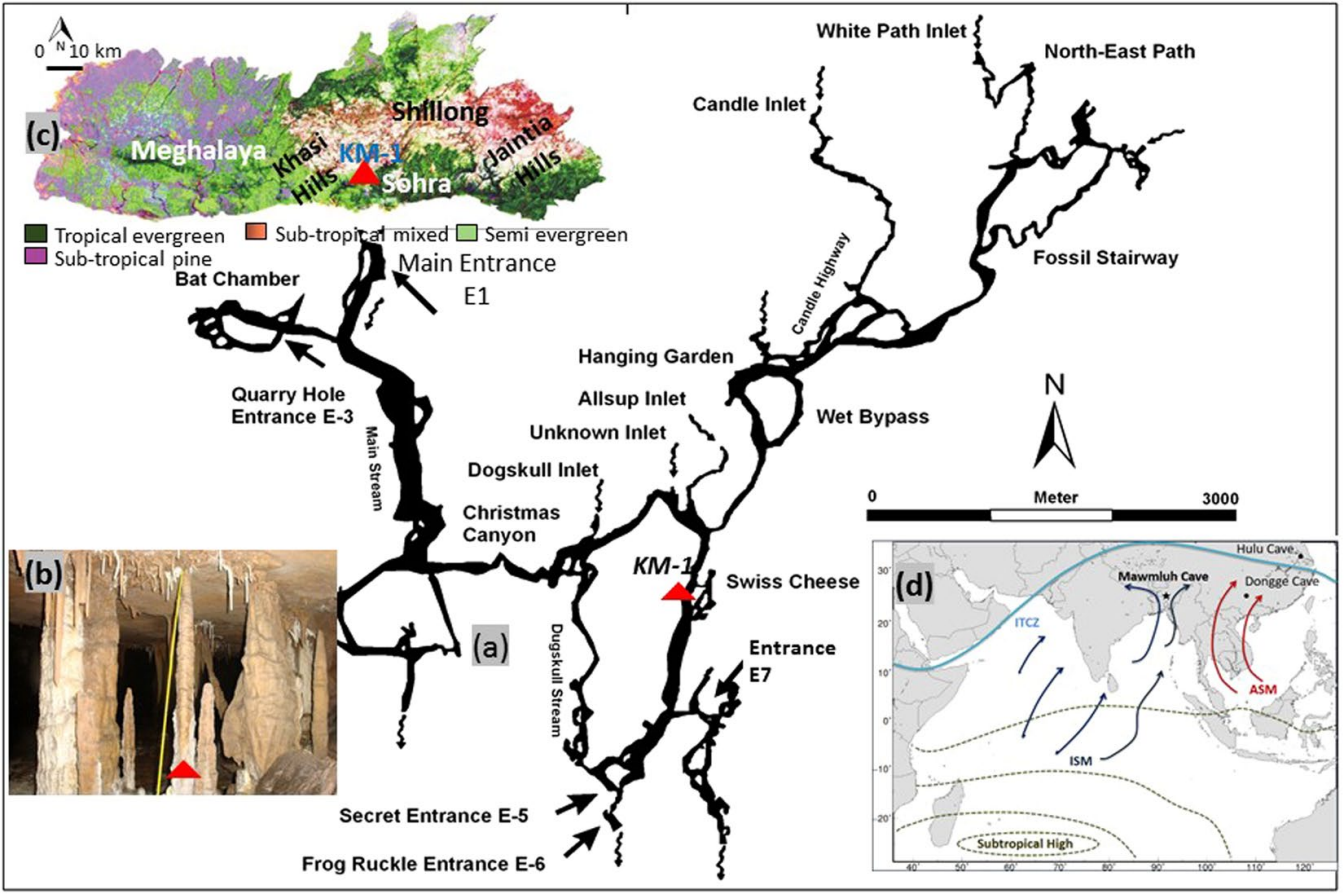
Mawmluh cave and surroundings: (**a**) map of Mawmluh cave system (plan view)^32^; (**b**) photo from the KM-1 stalagmite inside the Mawmluh cave system; (**c**) physiography and dominant vegetation pattern in Meghalaya^36^; (**d**) location of Mawmluh cave as well as Hulu and Dongge caves that were used for comparison. Light blue line indicates the Intertropical Convergence Zone (ITCZ) during the summer monsoon. Indian summer monsoon (ISM) originating in the Bay of Bengal influencing rainfall in the study area is indicated by dark blue arrows. The Asian summer monsoon (ASM) impacting eastern China is indicated with red arrows^25^. Red triangles in (**a**), (**b**) and (**c**) indicate Krem Mawmluh (KM-1) stalagmite.

During summer, the Indian continental landmass heats up rapidly and results in the development of a low air pressure mass that blows moisture led winds from the ocean towards the continent, and results in heavy rainfall (Fig. 1). The British, a colonial power in South Asia during the 1800s, referred to this seasonal heavy rainfall in India extending from June to September as ‘monsoons’. Even small variability in rainfall across the Indian sub-continent, where monsoons account for ~80% of precipitation, has great impacts on the socio-economic conditions of people who largely depend on agriculture. For example, as recently as the late 1960s, El Niño driven failure in monsoon precipitation for three consecutive years resulted in >1.5 million deaths in India from droughts that caused failed crops and famines^1^. In particular, the Intergovernmental Panel on Climate Change suggests extreme changes in the southwest monsoon output, its pattern and distribution/intensity in southeast Asia^2^, which poses grave concern for the socioeconomic structure in this region.

While early prediction can provide widespread relief, modeling and accurate projection of monsoon intensity continue to be challenging, both on local as well as regional scales. This is due to the limited historical and instrumental records on centennial-millennial scale, and high variability in monsoon intensity. The longest instrumental record of monsoon precipitation dates back to nearly 150 years^3^, which falls far too short to reveal long-term precipitation changes. Hence, studies based on various physical and chemical proxies in a range of different climate archives such as lakes, rivers, peat deposits, and stalagmites retrieved from the core monsoon zone have been investigated to trace the variability in southwest monsoon. Changes in the intensity within the core monsoon zone have been assessed using grain-size variation, pollen assemblages^4,5^, δ^13^C shifts in vegetation^6^, diagnostic biomarkers^7^ and δ^18^O shifts^8–10^. Notably, comparison of these different paleoclimate records indicates that periods of strongest and weakest monsoon winds were not always coupled to wettest or driest periods^4^. This is because monsoon precipitation is not entirely limited by moisture supply through the cross-equatorial flow, but also number of other factors that determine the actual over-turning of available moisture. The simultaneous correlation between monsoon driven wind circulation and its intensity (implying monsoon precipitation) is found to be 0.30 for July and 0.22 for August based on a sixty year (1901–60) data set^11^ suggesting other sources of variance that affect the monsoon intensity in south east Asia.

## Combining proxy records in stalagmites

Stalagmites provide high-resolution paleoclimate records describing both local and global environmental conditions in terrestrial environments^12–16^. Stalagmites are well suited for U-Th dating, and their growth may respond to climate driven changes, particularly during the high amplitude glacial-interglacial shifts. Thus, growth rate in stalagmites, although not necessarily, is often found to be proportional to the availability of water dripping inside the cave that results in enhanced speleogenesis^13^ during wet periods, and vice versa. In addition, various geochemical proxies such as stable C and O isotopes, mineralogy (calcite-aragonite), trace metals, and more recently biomarkers have been used in stalagmites to reconstruct the monsoon intensity, paleotemperature or paleovegetation cover^12,13,17,18^ (Table 1). The δ^18^O signal is most commonly reported in stalagmites^14^, and it is primarily controlled by precipitation that varies inversely with the amount and fraction of water vapor removed from clouds originating in the open oceans^9,10^. Hence, an increase in δ^18^O signal has been interpreted as a shift towards an earlier withdrawal of the monsoon, and a general decline in the total amount of precipitation within the core monsoon region^9,19,20^. The δ^13^C values in C_3_ plants is controlled by atmospheric *p*CO_2_ and its values in speleothems are therefore related to climatic conditions because they provide a first-order control on soil productivity and vegetation type^21^. Hence, organic matter preserved in speleothems could reveal changes in δ^13^C values spanning the glacial-interglacial cycles and vegetation cover. In particular, the covariance noted between δ^13^C with δ^18^O signals in speleothems indicates climate driven vegetation changes, and increase in the vegetation cover is reflected as depleted δ^13^C and δ^18^O values in speleothems^22^. In this context, the role of microbial input to soil organic matter^23^ affecting the vegetation signal preserved in δ^13^C of the speleothem should be comparatively minimal in caves covered with thin soil cover.

**Table 1:** Summary of major environmental conditions indicated by δ^13^C, δ^18^O, and TEX_86_. See Fig. 2 for details.

Period	δ^13^C	δ^18^O	TEX_86_
Last Glacial Maximum	relatively dry (↑)	weak ISM (↑)	rel. cold
End of Glacial Maximum	relatively dry (↑)	weak ISM (↑)	coldest
End of glacial	relatively dry (↑)	very weak ISM (↑)	cool
Bølling-Allerød	relatively dry (↑)	—	cool
Younger Dryas	very dry (↑↑)	very weak ISM (↑↑)	cool
Holocene	wet (↓↓)	strong ISM (↓↓)	warm

for δ^13^C: ↓ - depleted value refers to increased vegetation cover and vice versa.

for δ^18^O: ↓ - depleted/low/more negative value refers to high rainfall and vice versa.

Krem Mawmluh (KM; Krem means cave in the local Khasi language) located in Meghalaya in northeastern India has been investigated to trace the variability and strength of Indian Summer Monsoon (ISM) based on the δ^18^O record^24–26^. The strong seasonality effect on the isotopic composition due to the ISM is retained in drip water carbonates^25^, that precipitate to form stalagmites, and allow reconstruction of high-resolution climatic records. The δ^18^O records indicate abrupt changes in ISM during the warm Bølling-Allerød and early Holocene periods contrasting the weakening that occurred during the cold Heinrich events and the Younger Dryas^24,26^. Alterations in temperature gradients affected wind circulation patterns over the Bay of Bengal and northeast India that ushered these changes. However, uncertainties in the ISM variability persist, particularly the intensity and extent of such changes as well as their impact on landscape on both local and regional scales.

Glycerol dialkyl glycerol tetraethers (GDGT), which relate to environmental variables and, in particular, temperature^27^ were first reported in speleothems in 2011^28^. Since then, it has been reported that the majority of GDGTs in stalagmites are derived from *in situ* microbial communites within the cave or vadose zone^28–30^. Based on the distribution of these compounds, researchers^31^ proposed worldwide speleothem based calibration equations for TEX_86_-derived surface mean annual air temperature MAAT (R^2^ = 0.78, standard error ± 2.3 °C) and TEX_86_-derived cave MAAT (R^2^ = 0.68, standard error ± 2.0 °C) providing a benchmark for GDGT based paleotemperature reconstruction in speleothems (see Table 1, Tables S1 and S2, equations S1 and S2, supplementary data).

Both stalagmite GDGT reconstructions and their validation by comparison with detailed δ^18^O records are so far lacking making it difficult to assess the usefulness of these organic biomarkers in paleoclimate reconstructions. Bridging this gap, the present study evaluates if GDGT based indices for paleotemperature estimation can contribute towards a better understanding of past ISM variations. While there is little doubt that GDGTs can provide reliable climate signals in various sedimentary environments (e.g. lakes, peats, and soils)^27^, this is the first paleoclimatic reconstruction attempted with these compounds in a stalagmite supported by other complimentary high-resolution climatic signals. We correlated the GDGT signals in a stalagmite from Krem Mawmluh (KM-1) with the δ^18^O and δ^13^C records, Sr/Ca ratio and stalagmite growth rate to address the sensitivity of these organic compounds to climate change transcending the glacial-interglacial continuum.

## Study area

Krem Mawmluh is located on the southern fringe of the Meghalaya Plateau (25°15′32″N and 91°42′45″E; Fig. 1) in Khasi Hills near Sohra (Cherrapunji). The cave was first reported by Oldham in 1859^32^. The Khasi Hills are an uplifted Precambrian crystalline complex and form the northeastern extension of the Indian Peninsular Shield. This region is characterized by the dominance of southwest monsoons receiving abundant rainfall (~12,000 mm/yr) during the months of June to September^33^. However, rainfall in recent years has steadily decreased to ~8000–9000 mm/year. The orographic rain results from clouds originating in the Bay of Bengal that drifts towards the Bangladesh plains (Fig. 1). These clouds hit the Khasi and Jaintia Hills and rapidly rise to the upper atmosphere, where they swiftly cool down, and result in heavy precipitation^33^. Despite the heavy summer rainfall, this region suffers from water shortage during rest of the year^34^ due to poor water management strategies.

The cave is about 7.1 km long and developed as a complex sub-horizontal maze of passageways (Fig. 1) along the contact between the 9-m thick dolomitic Lakadong member of the Sylhet Limestone and the Therria Sandstone (both Lower Eocene in age^35^). The cave is overlain by 30–100 m thick and heavily karstified host rock consisting of limestone, sandstone, and a 40–100 cm thick coal layer. The terrain in and around Cherrapunji has a thin soil cover consisting of undulating grassland with pockets of shrubs, bushes and forested areas^36^. The poor soil cover (due to erosion) and rocky sub-surface is not favorable for vegetation despite the heavy rainfall.

The main entrance located at 1,160 m above sea level has partly caved in (there is extensive limestone mining which involves blasting the mountain sides with explosives that has damaged many caves). However, additional entrances exist through dolines into Krem Mawmluh (Fig. 1a). The secret entrance E-5 offers a more suitable path leading into a large chamber above the Gold Fish Pond, which remains isolated from floodwaters that fill large sections of the cave during heavy rainfall. In this large chamber, the 87-cm long KM-1 stalagmite was retrieved. The stalagmite was sectioned along its growth axis, and sampled for various analyses to establish the chronology, stable C and O isotope trends, trace elements, and GDGT signatures (see supplementary data).

## Results and Discussion

### Millennial-scale climate variability

StalAge^37^ modeling of twelve ^230^Th dates in stratigraphic sequence indicated the age in KM-1 to extend from 22.7 ka (Last Glacial Maximum; LGM) to 6.6 ka (mid-Holocene) covering a period of ~16,000 years (2σ error of 0.18–1.1 ka; see supplementary data and Fig. F1). The modeled ages reveal a drastic change in KM-1 growth rate with the onset of the Holocene interglacial climate following a dramatic peak in growth rate during the Younger Dryas (YD; Fig. F2 supplementary data).

**Figure 2:**
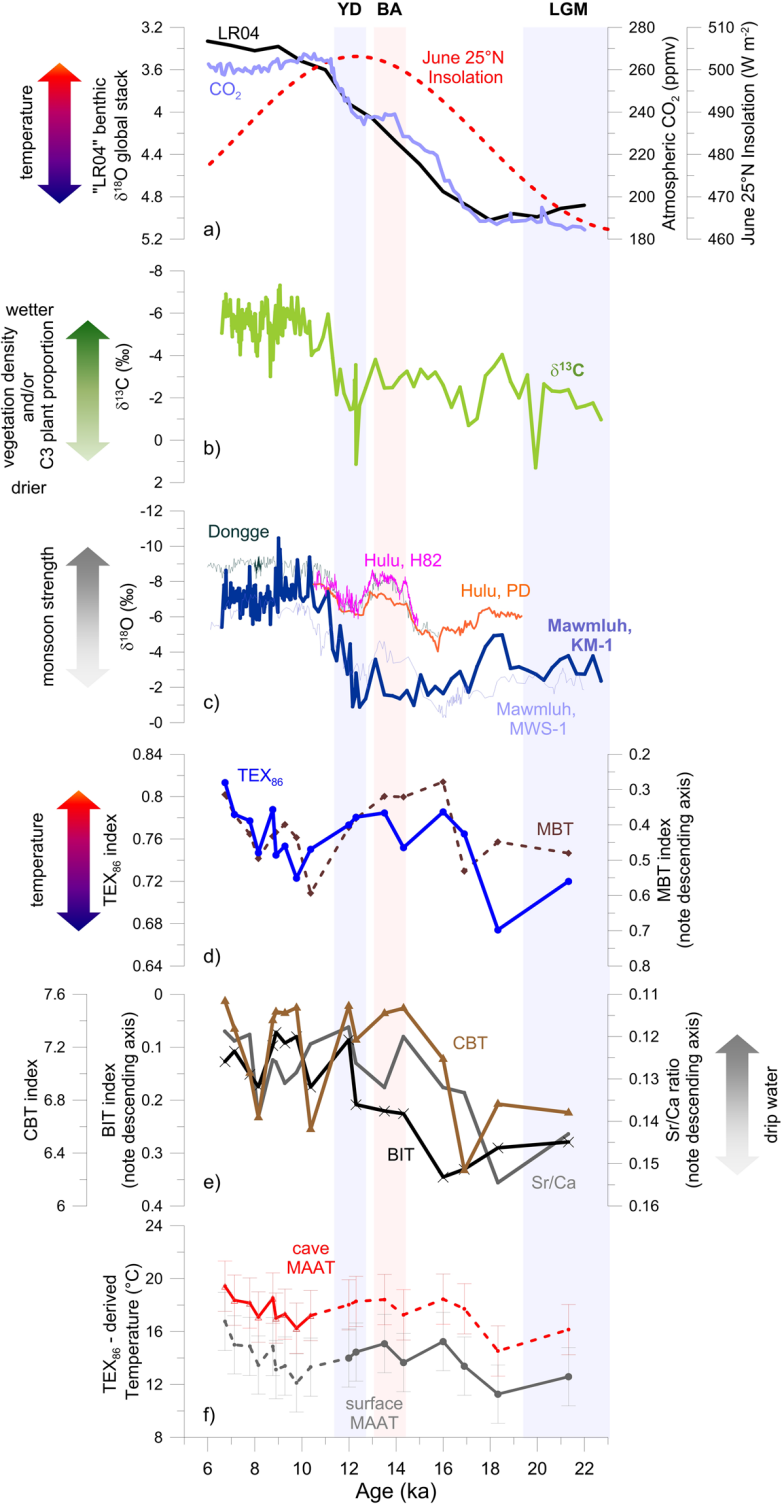
Climate reconstruction in Mawmluh Cave over the 22 to 6 ka period in the stalagmite KM-1, and other relevant caves in the region. (**a**) “LR04” benthic δ^18^O stack (black line^59^) as reference for global climate changes; June insolation at 25° N (dotted red line)^60^; and global atmospheric CO_2_ evolution in EPICA Dome C ice (purple line)^61^; (**b**) δ^13^C in KM-1 (green line) and stalagmite KM-1 growth rate (dashed turquoise line); (**c**) δ^18^O in speleothems from Mawmluh cave, i.e. KM-1 (thick deep blue line) and MWS-1 (thin blue line^20^), as well as Dongge Cave (dark green line^16^), Hulu Cave (stalagmite H82, pink, and stalagmite PD, orange^62^); (**d**) GDGT-based TEX_86_ (blue line) and MBT (dashed dark brown line) indices in stalagmite KM-1. TEX_86_^63^ and MBT^49^ were calculated as indicated in Table S1; (**e**) Sr/Ca ratio (grey line), BIT index (black line) and CBT index (brown line) in KM-1; (**f**) TEX_86_-derived paleo-temperature for surface MAAT (Table S2, equation S3) and cave MAAT (Table S2, equation S4). Both equations refer to the calibrations for sample sets with BIT values ≤ 0.4 only^31^. The error bars refer to the standard errors of the temperature estimates (i.e. 2.2 °C for surface MAAT calibration and 1.9 °C for cave MAAT calibration)^31^. Data are from this study unless stated otherwise.

The KM-1 stalagmite contains 170 δ^18^O and δ^13^C measurements with overall temporal resolution of ~95 years for the period 22.7 ka to 6.6 ka (Fig. 2b,c). With increased growth rate during the transition from YD to the early Holocene, the sample resolution increases to ~29 years, whereas during the glacial period the resolution is as low as 335 years at 0.5-cm interval. Owing to the so called “amount effect”, i.e. the relationship between δ^18^O and precipitation amount^38^, the δ^18^O value in speleothems from tropical India has been related to the Indian monsoon intensity in previous studies^9,24,39^. Further, stable isotope time-series from the northeast Indian speleothems capture the shift in moisture source and transport pathway, as well as the isotopic composition in the Bay of Bengal surface waters, all of which in turn reflect the ISM strength^24,40,41^. In the KM-1 stalagmite, δ^18^O values range from −9.74‰ to −0.18‰ (Fig. 2c). The mean δ^18^O value for the Holocene (−6.49‰) was 4.70‰ more depleted than that for the glacial period (−1.79‰; Fig. 2c). It is evident from the KM-1 record that deglaciation started after ~18 ka with increase in global temperature and atmospheric CO_2_. However, this short-lived deglaciation was punctuated by the cold Heinrich 1 (H1) event at ~17 ka. The low δ^18^O values at ~15 and ~13.5 ka, refer to increased monsoon strength around the Bølling-Allerød. However, owing to the low resolution (~335 years), these events are not fully resolved. The period 12.1 ka to 11.1 ka, also witnessed pronounced transition from glacial to the wetter and more humid Holocene; the δ^18^O value decreased drastically from −0.18‰ at 12.1 ka to −6.82‰ at 11.1 ka and stayed close to it for most of the Holocene. The temporal resolution increases abruptly during this period due to sharp increase in growth rate (Fig. F2, supplementary data) allowing us to consider short-lived abrupt departures in the record, which was not possible for the glacial period.

The stable carbon isotope (δ^13^C) composition is more ambiguous because a range of climatic factors, including source change, ventilation, drip rate, temperature, and moisture affects it^12,13,25^. Mainly used as a proxy for C_4_ versus C_3_ plant types, δ^13^C is also influenced by processes such as abiotic fractionation in the cave or microbial mediated fractionation within the overlying soil. The δ^13^C record for KM-1 (Fig. 2b) ranges from 1.95‰ at 19.9 ka to −6.61‰ at 9.07 ka. The δ^13^C profile for the Holocene is on an average 3.42‰ more depleted than the glacial period. During periods of high vegetation density, the contribution from soil *p*CO_2_ may increase drastically, that typically imparts a more depleted δ^13^C signature in the speleothem carbonate compared to that derived from bed rock dissolution^25^. The δ^13^C data reveal that the vegetation density would have increased enormously with advent of the wet Holocene epoch where it records ~−4‰ shift on an average. It is suggested that a decrease of ~2‰ in plant δ^13^C values from LGM to Holocene, contributes towards a major *p*CO_2_ controlled depletion in speleothem δ^13^C^13^. The authors suggest of a ~70% vegetation driven change in δ^13^C values on the glacial-interglacial scale. This apparent change points to the fact that a ~30% decline in δ^13^C may be incorporated by enhanced drip-rate during the Holocene. The δ^13^C profile in KM-1 reveals the maximum values at 20 ka and 12.3 ka BP (1.95 and 1.80‰, respectively). These high δ^13^C values correspond to the LGM and YD and reflect that vegetation in the area reduced greatly during these cold periods. A combined effect of low drip rate and low cave air *p*CO_2_ led to an increase of δ^13^C as suggested earlier^25^. However, we do not rule out a slight bias in the KM-1 data since these extreme events are represented by single data points (due to low sampling resolution covering these periods). During YD and LGM, rainfall was extremely low as evident from the δ^18^O and KM-1 growth rate. The low drip rate may have resulted in longer residence time leading to precipitation of calcite, and thereby higher δ^13^C. Consistent with this hypothesis, a study focusing on two sites with different drip rates indicate ~3‰ increase in δ^13^C that coincided with lower drip rate at the site^42^. Thus, δ^13^C values in KM-1 refer to changes in vegetation cover, density and primary vegetation sources related to monsoon intensity that varied during this period and serves as a simple analog. Notably, there is a very high correlation between δ^18^O and δ^13^C values (R^2^ = 0.75, p < 0.05), which implies an overlapping climatic control on the isotopic signals similar to other studies^21,22^.

### Glycerol dialkyl glycerol tetraethers trends

We used bacterial GDGTs (br-GDGT) and archaeal GDGTs (iso-GDGT) that allowed the calculation of various paleoclimatic indices in the KM-1 stalagmite. The general trend in TEX_86_ index follows the glacial-interglacial transitions observed in the isotope records (Fig. 2d). The highest TEX_86_-derived temperatures reconstructed using the cave MAAT calibrations^31^ (Table S2, equation S4, supplementary data) in our record were observed at ~6 ka, reaching 19 to 20 °C (±1.9 °C standard error of the estimate; Fig. 2), which is close to the modern surface and cave MAAT of 19 °C^25^. The global cave MAAT calibration is based on a small number of samples (*n* = 16 from 6 sites)^31^, and thus reconstructed values should be carefully interpreted until a revised calibration including a considerably larger data set is available.

Rapid transitions are also observed to be coupled in the isotope and GDGT records. For instance, a spike in TEX_86_ at ~9 ka is followed by a sharp decrease that coincides with a decrease of ~3‰ in δ^18^O values; TEX_86_ again increased around 8 ka (Fig. 2c,d). This agreement points towards a close relationship between the paleo-temperature and hydrology. Some important discrepancies, however, indicate shifts in the local climatic system. For instance, the TEX_86_ increases as early as ~18 ka, following closely the 25 °N summer insolation until around 10 ka (Fig. 2a,d). Such early initiation of the deglacial warming has been reported in the nearby Bay of Bengal (supplementary data Fig. F2), Arabian Sea and eastern Indian Ocean^41,43^. The isotopes, in contrast, remain steadily low until the YD at ~12 ka, lagging the insolation and consequent deglacial warming by ~6 ka (Fig. 2). Likewise, a 3 ka delayed East Asian summer monsoon intensification compared to East Asian air temperature had been reported previously^44^. In KM-1, the isotopic signatures are clearly divided between the last glacial (pre-12 ka) and the Holocene (post-12 ka), but the TEX_86_ index does not follow this division strictly (Fig. 2d). While, the TEX_86_ is positively related to the isotopic signatures within the respective glacial and Holocene subsets it is not for the entire record (supplementary data Fig. F3). This discrepancy could be caused by: (a) a local driving factor that decouples the temperature from southwest monsoon, and/or (b) shift in the hydrological system leading to a change in the potential GDGT sources.

A shift in driving force that leads to a disparity between the temperature and ISM strength is the possible decoupling of Northern Hemisphere insolation and ocean circulation pattern caused by disintegrating ice sheets. For instance, the YD is a markedly cold event during which temperatures across the Northern Hemisphere dropped considerably, even though the Northern Hemisphere insolation was at peak (Fig. 2a). The YD is attributed to the fresh water influx into the North Atlantic that eventually resulted in the weakening of the Atlantic meridional overturning circulation (AMOC) and weakening of the monsoon^45,46^. While a sharp decline in δ^13^C during the YD alongside a slower drop in δ^18^O reflect such weakening of the ISM and rapid shift to dryer conditions during the YD, the TEX_86_-index follows instead the increase in 25 °N summer insolation until around 10 ka (Fig. 2). Thus, in contrast to the monsoon and hydrological regime, the continental air temperature does not seem to be directly affected by the slowing of the AMOC. This is consistent with modern observations that suggest warm and cold phases associated with the Atlantic Multi-decadal Oscillation have no effect on the continental air temperature in the Indian sub-continent^47^.

After ca. 10 ka, the δ^13^C and δ^18^O indicated a shift towards wetter conditions associated with a strong monsoon from as early as ~11.5 ka, and the TEX_86_ began to increase at ~10 ka, indicating a shift towards warmer temperatures. During this most recent period in our KM-1 record, characterized by strong monsoon, wet conditions and warmer temperatures, the insolation steadily decreases (Fig. 2). Elevated temperatures during the Holocene despite decreasing local insolation might have been caused by positive feedback factors, such as atmospheric CO_2_ concentrations, that are consistently high between 10 and 6 ka (Fig. 2). The decoupling of monsoon strength and air temperature during early glacial stages have been related previously to the impact of CO_2_ levels coupled with Southern Hemisphere processes in East Asia^48^. In addition, source change in biomarker deposition driven by changes in the hydrological system discussed in the following section, might also play a role explaining the decoupling between the ISM strength and temperature.

A third fundamental issue that might lead to the observed decoupling between GDGT and isotope derived signals, is the low-resolution of GDGT analyses. The GDGT analysis is constrained by the large amount of sample required to dissolve the carbonate fraction to extract the sufficient quantity of lipid biomarkers. This drawback will always constrain the generation of high-resolution biomarker data compared to the stable isotope records in speleothems that are almost always low in organic C content. Nevertheless, GDGTs provide a quantitative estimate of temperature, and serve as an important complementary proxy to the stable isotope records that fall short despite the high analytical precision.

### Indicators of shifts in GDGT sources and hydrology

The TEX_86_ index is mostly used in aquatic environments due to the predominantly aquatic provenance of iso-GDGTs. In contrast, the br-GDGTs are mainly produced by soil thriving organisms and are used to calculate the Methylation Index of Branched Tetraethers (MBT) and the Cyclisation of Branched Tetraether (CBT) indices^49^. The MBT is negatively related to the TEX_86_ (Fig. 2e), while the CBT index is not related to the TEX_86_ values over the 22 ka glacial-interglacial period illustrated in KM-1. Applying published calibration equations for MBT or MBT/CBT-derived MAAT (Table S2, equations S5–S14)^31,49,50^, decreasing MAAT after 10 ka is observed, leading to very low most recent temperatures. Applying the MBT/CBT-derived MAAT calibration for cave MAAT (Table S2, equation S12, supplementary data)^31^ results in most recent temperatures around 12 °C (± 2.5 °C standard error of the estimate; supplementary Fig. F2). This is much lower than the iso-GDGT derived temperatures and measured modern temperatures^25^. In lakes, the cold bias of br-GDGT derived MAAT has been proposed to be a result of varying contributions of terrestrial and *in-situ* production^51^.

In cave systems, both iso- and br-GDGTs may originate *in-situ*, i.e. inside the cave, but input from the overlying soil cover cannot be excluded, and its extent might vary with the hydroclimatic conditions and time. The br-GDGT concentrations in soils along an altitudinal transect from this area are up to 15 µg/g dry weight of soil similar to other humid tropical soils^52^. Likewise, the iso-GDGTs have been reported in detectable amounts in soils from various regions of the world^53,54^. Hence, in principle, a soil-derived source for br- and iso-GDGTs are plausible in the Meghalayan soils. However, the soil layer overlying the Mawmluh cave is poorly developed and occurs as a thin veneer^36^ making a major soil origin unlikely in this particular case. The first insight into potential sources of speleothem GDGTs in the Heshang Cave, China^28^ compared the distribution of br- and iso-GDGTs in cave speleothems with the distribution in overlying soils. They found that the br-GDGTs dominated over iso-GDGTs in soil, whereas iso-GDGTs prevailed over br-GDGTs inside the cave. Based on the GDGT distributions, the authors concluded that both iso-GDGTs and br-GDGTs were most likely produced *in-situ*^28^. Likewise, another study concluded in a recent review that most GDGTs are probably sourced from the cave itself^29^. Hence, iso- and br-GDGTs in KM-1 might also have a predominant *in-situ* provenance, with potential influence from soil microbes, especially br-GDGTs from the soil-thriving bacteria. This impact could vary with changes in the hydrological regime, and lead to some of the decoupling observed between the reconstructed temperature and monsoon strength.

A major hydrological shift is supported by both the Sr/Ca ratio (Fig. 2e) and br-GDGT-derived CBT index. Briefly, a higher Sr/Ca ratio indicates drier conditions, low flow, inducing longer residence times, i.e. longer time for water-soil and/or water-rock interactions, and degassing of CO_2_ into air pockets and calcite precipitation, typically leading to enrichment of Sr and/or removal of Ca. During wetter conditions the residence time would be shorter and CO_2_ will degas slowly, resulting in reduced calcite precipitation^55^. The CBT index has typically been used to derive pH values in soils (supplementary Table S2, equation S15)^49^. However, a recent study indicated a correlation between CBT and soil moisture content in Chinese loess, and absence of correlation with pH^56^. Both, soil moisture and pH might be indirectly related though, as soil pH is often affected by rainfall. CBT-derived pH values^49^ range from 6.8 to 8.3 and water content^56^ varies between 10 to 30% with lowest pH and water contents observed during the late glacial period and highest after ca. 15 ka BP. The low Sr/Ca ratio and higher CBT-derived water content (or pH) during the Holocene are accompanied by higher br- and iso-GDGT abundances (supplementary data Fig. F2). The Holocene apparently offers favorable conditions, e.g. more drip water, for GDGT producing organisms.

An indication of environmental shifts within the Mawmluh cave is further provided by the BIT index (Fig. 2e), which is positively correlated to δ^18^O (R^2^ = 0.49; p < 0.005) and δ^13^C (R^2^ = 0.33; p < 0.05; supplementary Fig. F3) in KM-1. The BIT index reflects the relative contribution of iso-GDGTs produced by Thaumarchaeota compared to the br-GDGT produced by bacteria. In KM-1, the BIT values in general are relatively low for a continental site (BIT < 0.35 out of a range varying from 0 to 1) supporting the prevalence of iso-GDGTs. Typical BIT values in soils are higher than 0.3^54,57,58^ supporting our assumption of predominant *in situ* provenance of GDGTs in Mawmluh cave. In KM-1, the BIT is highest and closest to typical soil BIT values (0.35; Fig. 2e) during the late glacial, which indicates a lower relative contribution of Thaumarchaeota and possibly a higher contribution of soil derived GDGTs. The BIT values drop to around 0.10 during the Holocene (Fig. 2e), which indicates a higher relative contribution of Thaumarchaeota and possibly less contribution from soil-derived GDGTs. This drop in BIT occurs at a time of recovery towards wetter and stronger monsoons following the very dry and weak period at the end of LGM (Fig. 2).

**Figure 3:**
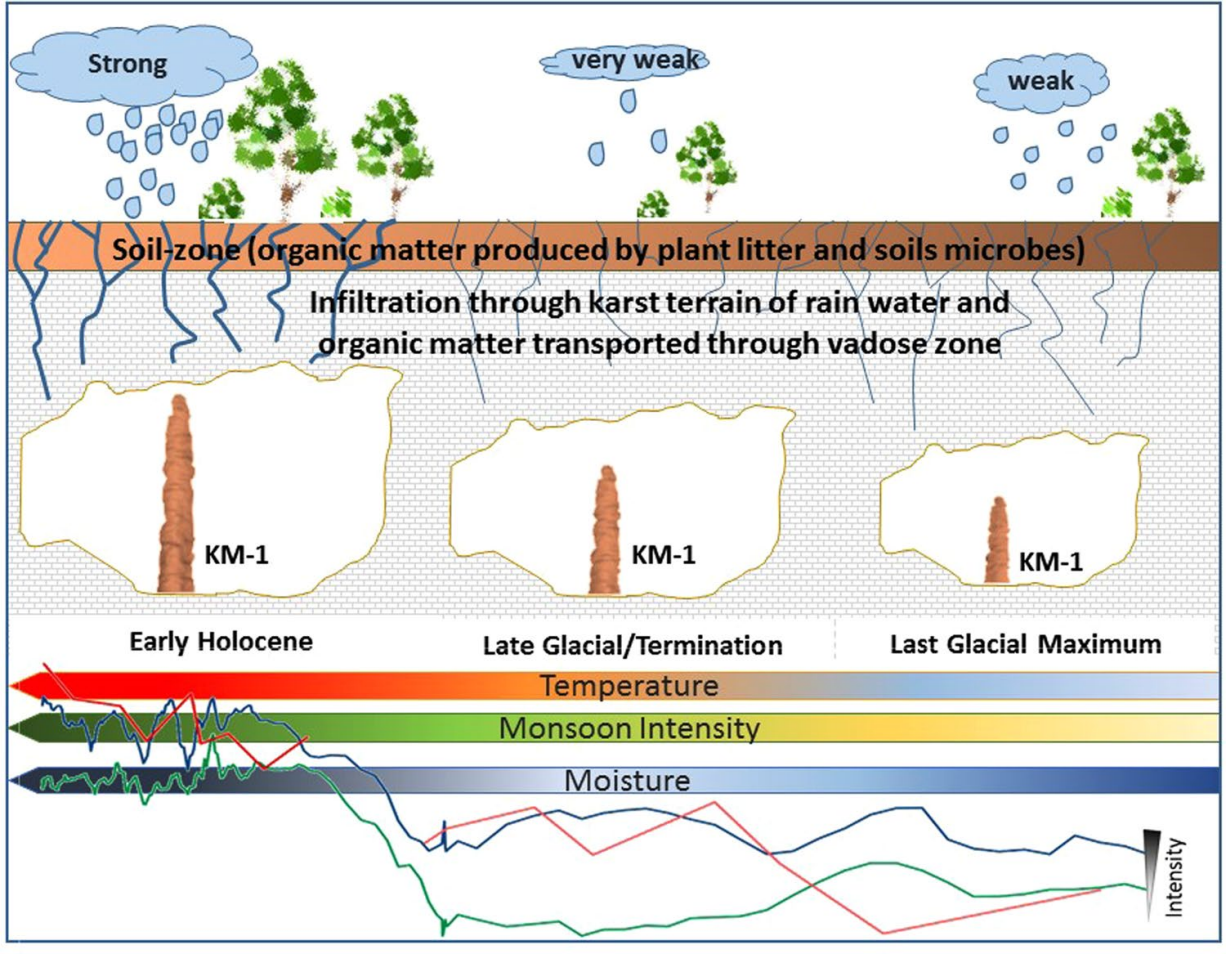
A conceptual figure tracing the formation of speleothems in a karst terrain, such as Mawmluh Cave. Rainwater absorbs CO_2_ from atmosphere and infilters through the soil-zone. The low pH dissolves surrounding limestone forming a karst terrain; *p*CO_2_ conditions, calcium bicarbonate saturation and humidity results in precipitation of CaCO_3_ out of drip water forming stalagmites inside the cave. As the stalagmite grows over time as shown in Krem Mawmluh, it records faithfully based on different proxy measurements (δ^18^O, δ^13^C and GDGT indices): moisture ( blue line), temperature ( red line) and monsoon intensity ( green line) helping us to quantitatively reconstruct with greater precision the paleoclimate changes associated with monsoonal fluctuations spanning from the last glacial maximum to early Holocene. The monsoonal fluctuations ushered increased warming and moisture/soil infiltration, and increased growth rate in stalagmites forming inside the cave.

A schematic representation of speleogeneis in Krem Mawmluh is shown in Fig. 3. The figure conceptualizes how temperature and monsoon fluctuate across the glacial-interglacial continuum bringing forth distinct changes in stalagmite growth, and fluctuations in temperature and the moisture driven proxies. The stable isotope trends, inorganic geochemical, and GDGT-derived proxies - all support the assumption of a major shift in paleo-hydrological conditions at the end of the LGM. Such hydrological shifts may affect the provenance of the GDGT-derived temperature signals. A weak monsoon period resulting in low drip water rates may limit GDGT production by microbial communities living inside the cave and reduce the relative contribution of *in situ* production. In contrast, the slow infiltration may increase the residence time in the overlaying soil or karst system and increase the relative contribution of GDGT production by “surface” (i.e. soil or karst) microbes. On the other hand, wet and humid conditions during high monsoon intensity increase drip water rates and result in increase of in-cave production. A fast flow, in contrast, lowers the residence time in the overlaying soil or karst system, and lowers the relative contribution of GDGT production by “surface” microbes. In this scenario, the paleo-temperature reconstruction during the last glacial (pre-12 ka) would represent a mix between surface air temperature and the *in-situ* cave temperature, with an increasing contribution of cave temperature signal since the onset of the Holocene (post-12 ka). This shift in signal source could explain the good correlation in the pre-glacial and post-glacial subsets between TEX_86_ and isotope signals (supplementary Fig. F3), but a general lack of good fit over the entire record (22 ka to 6 ka). The slight decrease in δ^13^C from ~17 ka onwards that is not observed in the KM-1 δ ^18^O record indicates a strengthening of the monsoon activity, and possibly, the amount of drip water. This would lead to TEX_86_ representing cave temperature since the end of the last glacial period. Figure 2f shows TEX_86_-derived temperature reconstructions assuming a different source by using the equation for surface MAAT^31^ (supplementary Table S2, equation S3), and using equation for cave MAAT (supplementary Table S2, equation S4). Assuming a predominantly surface signal for the glacial period the pre-12 ka temperatures range from around 11 °C to almost 17 °C, while the Holocene temperatures (post-12 ka), assuming a predominant *in-situ* provenance range from around 16 °C to almost 20 °C (Fig. 2f). These reconstructed temperatures fall within the upper range of modern air temperatures observed in the region (4 to 35 °C) and inside the cave (6.8 and 23.5 °C)^25^.

## Conclusions

The stalagmite KM-1 spans the last two contrasting climatic regimes and gives us a unique opportunity to consider the last glaciation, its transition into Holocene and the period covering early to mid-Holocene in the Indian sub-continent. We observed several rapid shifts in δ^18^O and δ^13^C isotope values, for example during the LGM and YD. Over the 16-ka record, a progressive increase in monsoon strength was coupled to higher TEX_86_ temperatures from the LGM to Holocene. However, temporal mismatches indicate a decoupling of the local temperature from the monsoon strength in the cave and its surroundings, which is not apparent from the isotopic record alone. Insolation and atmospheric CO_2_ levels might both play an important role in this decoupling. A further key role might be shifts in hydrology and the source of these signals. In KM-1, the GDGT-derived indices seem to provide both temperature and moisture information that can be disentangled by using different calibrations published for different scenarios. This approach has proven major hydrological shifts in KM-1 at the end of LGM, and before the onset of the Holocene. Thus, this study proves that GDGTs are a valuable addition to paleoclimate studies in speleothems. They can provide complementary information to isotope records and provide valuable insights into monsoon variability and its interpretation.

## Electronic supplementary material

Supplementary information
