# Supplementary material for: Temperature and Monsoon Tango in a Tropical Stalagmite: Last Glacial-Interglacial Climate Dynamics
Source: Sci Rep. 2018 Mar 29;8:5386. doi: 10.1038/s41598-018-23606-w (PMC5876336; doi:10.1038/s41598-018-23606-w)
Supplement: Supplementary file 1 — Supplementary information [file 41598_2018_23606_MOESM1_ESM.pdf]

Supplementary data

Temperature and Monsoon Tango in a Tropical Stalagmite: Last Glacial-Interglacial Climate Dynamics

Carme Huguet[1], Joyanto Routh[2], Susanne Fietz[3], Mahjoor Ahmad Lone[4], M.S. Kalpana[5], Prosenjit Ghosh[6], Augusto Mangini[7], Vikash Kumar[8], and Ravi Rangarajan[6]

[1] Departamento de Geociencias, Universidad de los Andes, Bogotá, Colombia
[2] Department of Thematic Studies, Environmental Change, Linköping University, 58183 Linköping, Sweden
[3] Department of Earth Sciences, Stellenbosch University, 7602 Stellenbosch, South Africa
[4] High-Precision Mass Spectrometry and Environment Change Laboratory (HISPEC), Department of Geosciences, National Taiwan University, Taipei 10617, Taiwan
[5] CSIR-National Geophysical Research Institute, Hyderabad 500007, India
[6] Centre for Earth Sciences, Indian Institute of Sciences, Bangalore 560012, India
[7] Institut für Umweltphysik, INF 229, Heidelberg 69120, Germany
[8] National Centre for Antarctic & Ocean Research, Goa 403804, India

Methods

Chronology

Dating was carried out in KM-1 using the U-Th method. Twelve samples were drilled along the growth axis at the Heidelberg Academy of Sciences, Germany. Dating was performed on a Finnigan MAT 262 RPQ mass spectrometer. The chemical blanks yielded less than 0.1 g for both ^{238}U and ^{232}Th indicating reliability of the extraction procedure and analyses. Specific details regarding the extraction procedure, detritus correction, and standards utilized for quantification are according to previous studies[1,2]. The age model was constructed using StalAge[1] (supplementary data Fig. F1). The model uses U-Th ages along with their associated errors and provides modeled ages with 95% confidence limits using a Monte-Carlo simulation.

Stable isotope analyses

Hendy test[3] was carried out at different depth on four distinct layers in KM-1, which confirmed the utility of the sample for further paleoclimatic studies. The results indicated that the sample formed under isotopic equilibrium. For stable C and O isotope analyses, samples were selected at every 0.5-cm interval. A Proxxon dental drill equipped with a 3-mm diamond tip drill bit was used to extract the samples along the growth axis. The spatially resolved carbonate samples were analyzed on a GasBench II peripheral coupled with MAT 253 Isotope Ratio Mass spectrometer. Carbonates were analyzed in batches of 16 sample entries along with three or more standards. Experimental protocol include preparation of CO_2 reacting carbonate powders with 105% phosphoric acid (MERCK) for a duration of 70 minutes (or more) at 70 °C bath temperature[4]. The inter-laboratory standard MAR-J1 is

considered as an internal reference standard that was calibrated with respect to IAEA standard NBS-19. Replicate analysis of NBS-19 calcite showed $\delta^{18}O$ and $\delta^{13}C$ reproducibility of ±0.08‰ and ±0.05‰, respectively.

Elemental analyses

For elemental analyses in the drilled sub-samples, acid (3M HNO_3) and milli-Q rinsed teflon vessels were used for sample digestion. Primary standards from Sigma-Aldrich were prepared gravimetrically using high purity Mg chips (99.99%), $CaCO_3$ powder (99.999%), and $SrCO_3$ powder (99.995%). Impurities of Mg and Sr from the $CaCO_3$ powder caused an insignificant bias of < 0.01% for Mg/Ca and Sr/Ca ratios in the primary standard solution. We measured the elemental concentrations using a Varian ICP-OES. The elemental concentration in our samples was compared to the standard SPS SW2 having the following concentrations (Ca 10 mg/l, Mg 2 mg/l, and Sr 250 µg/l). The reproducibility of these values in the standard was 1-3%, respectively. Sr measured in the solutions was up to two orders of magnitude lower than the standard.

Lipid analysis

Before drilling the stalagmite at different depths to collect the samples for biomarker extraction, the stalagmite pieces were thoroughly cleaned in deionized distilled water followed by dichloromethane to remove surface contaminants. About 10 g of sample powder was hand-drilled along the growth axis from 17 spots. The biomarkers were extracted from stalagmites following the adapted acid digestion method[5]. The stalagmite powder was digested with 3M HCl after introducing 20 ul of 500 mg/L deuterated-hexatriacontane (recovery standard) and reflux heated. The resulting solution after cooling was transferred into a separating funnel for extraction with dichloromethane (DCM; 30 ml x 5 times). The solvent extracted lipids were concentrated by evaporation under reduced pressure using the Buchi Syncore Analyst concentrator. The concentrated lipid extracts (1 ml) were reacted with 3 ml of 14% BF_3-methanol at 70 °C for 2 hrs. After cooling overnight at room temperature and the destruction of excessive BF_3 complex with ultra-pure deionized distilled water (3 ml), the methylated solution was extracted with hexane (2 ml x 6 times). The hexane extract was evaporated to near dryness under a high-grade nitrogen stream. The residue was further derivatized with 30 µl of BSTFA (N,O-bis (trimethylsilyl) trifluoacetamide) at 70 °C for 2 hrs in a sealed reaction vial and left overnight. The derivatized extract was taken to dryness under nitrogen and reconstituted in 150 µl of DCM for analyses of GDGTs and other biomarkers (e.g., alkanes, fatty acids, and sterols). A method blank was run with pure $CaCO_3$ to evaluate contamination resulting from extraction procedure.

The extracts were dissolved in hexane:isopropanol (99:1) and filtered through a 0.45 µm nominal pore size PTFE membrane previous to injection into a HPLC-MS (High pressure liquid chormatography-mass spectrometer) equipped with an autoinjector and an atmospheric pressure chemical ionization interface[6,7]. Compounds were separated using a CN column fitted with CN guard column (4.0 × 200 mm, 3 µm) with an increasing gradient from 99:1 (v:v) hexane:isopropanol to 1.8% isopropanol[6]. Detection of GDGTs was done in single ion monitoring (SIM) mode of $[M+H]^+$ ± 0.5 m/z units to increase signal-to-noise ratio[6]. Target protonated molecules were *m/z* 1302, 1300, 1298, 1296 and 1292 for iso-GDGTs and *m/z* 1050, 1048, 1046, 1036, 1034, 1032, 1022, 1020 and 1018 for br-GDGTs. The different GDGT compounds quantified in the lipid extracts were used to calculate ratios proposed in other studies (Table S1) to estimate the paleotemperature and prevalent signal source contributing to the GDGT pool.

Table S1 GDGT-derived indices and their basic description. Arabic numbers refer to number of rings in isoprenoid GDGTs published previously[8]. For instance, GDGT-1 has one moiety (mass-to-charge ratio (*m/z*) of 1300), GDGT-2 has two moieties (*m/z* 1298) etc. GDGT-5 refers to GDGT with five moieties, i.e. crenarchaeol. GDGT-5' refers to crenarchaeol regiosiomer. Roman numerals refer to chemical structures of branched GDGTs published previously[8]. Briefly, GDGT-I refers to br-GDGT with *m/z* 1022, GDGT-Ib refers to br-GDGT with *m/z* 1020, GDGT-Ic refers to br-GDGT with *m/z* of 1018, GDGT-II refers to br-GDGT with *m/z* 1036, GDGT-IIb refers to br-GDGT with m/z 1034, GDGT-IIc refers to br-GDGT with *m/z* 1034, GDGT-III refers to br-GDGT with *m/z* 1050, etc. The TEX_{86} index is based on the number of cyclopentane moieties of the iso-GDGTs[9]. The MBT is based on the degree of methylation, while the the CBT index is based on the degree of cyclization of the br-GDGTs[10] (Table 1). The BIT indicates the terrestrial (br-GDGTs) vs aquatic (crenarchaeol) contribution to the GDGT pool[11] (Table S1).

**Index**		**Calculation**	**Common application**	**Reference**
TEX_{86}	TetraEther indeX of tetraethers consisting of 86 carbon atoms	TEX_{86} = [(GDGT-2)+(GDGT-3) + (GDGT-5')]/[(GDGT-1)+(GDGT-2)+(GDGT-3)+(GDGT-5')	water temperature	[9]
MBT	Methylation Index of Branched Tetraethers	MBT = (I + Ib + Ic)/(I + Ib + Ic + II + IIb + IIc + III + IIIb + IIIc)	in combination with CBT: air temperature	[10]
CBT	Cyclisation ratio of Branched Tetraethers	CBT = - Log[(Ib + IIb)/(I + II)]	soil pH	[10]
BIT	Branched over Isoprenoid Tetraethers	BIT = (I + II + III)/(I + II + III + GDGT-5)	contribution of terrigenous relative to contribution of aquatic GDGTs to the total GDGT pool	[11]

Table S2. Published calibrations for temperature reconstructions based on indices derived from GDGT composition: TEX_{86}, MBT, MBT’, CBT. The calibrations were conducted against mean annual air temperature (MAAT) at surface or inside the cave.

**Equation number**	**Calibration equation**	**Description**	**Reference**
S1	MAAT (°C) = -7.4 + (33.3 x TEX_{86})	TEX_{86} vs surface MAAT equation 1	[8]
**S2**	MAAT (°C) = -7.3 + (32.2 x TEX_{86})	TEX_{86} vs cave MAAT equation 1	idem
**S3**	MAAT (°C) = -7.1 + (32.1 x TEX_{86})	TEX_{86} vs surface MAAT equation 2	idem, for sample sets where samples with BIT ≥0.5 are excluded
**S4**	MAAT (°C) = -9.2 + (35.2 x TEX_{86})	TEX_{86} vs cave MAAT equation 2	idem, for sample sets where samples with BIT ≥0.5 are excluded
**S5**	MAAT (°C) = (MBT – 0.122 – 0.187 x CBT)/0.02	MBT/CBT vs surface (top soil) MAAT	[10]
**S6**	MAAT (°C) = 0.81 – 5.67 x CBT + 31.0 x MBT’	MBT’/CBT vs surface (top soil) MAAT	[12]
**S7**	MAAT (°C) = 22.8 x MBT + 5.6	MBT vs surface MAAT	[8]
**S8**	MAAT (°C) = 21.9 x MBT + 4.3	MBT vs cave MAAT	idem
**S9**	MAAT (°C) = 0.3 x CBT + 12.3	CBT vs surface MAAT	idem
**S10**	MAAT (°C) = -5.8 x CBT	CBT vs	idem

	+ 10.8	cave MAAT	
**S11**	MAAT (°C) = 5.4 + (27.1 x MBT) – (4.8 x CBT)	MBT vs CBT vs surface MAT equation 1	idem
**S12**	MAAT (°C) = 6.06 + (21.3 x MBT) – (5.4 x CBT)	MBT vs CBT vs cave MAAT equation 1	idem
**S13**	MAAT (°C) = 6.0 + (20.9 x MBT) – (4.5 x CBT)	MBT vs CBT vs surface MAAT equation 2	idem
**S14**	MAAT (°C) = 6.6 + (20.2 x MBT) – (5.8 x CBT)	MBT vs CBT vs cave MAAT equation 2	idem
**S15**	pH = (3.33 – CBT)/0.38	CBT vs surface (top soil) pH	[10]

Figure F1 Modeled age in KM-1 stalagmite from Krem Mawmluh based on StalAge[1]

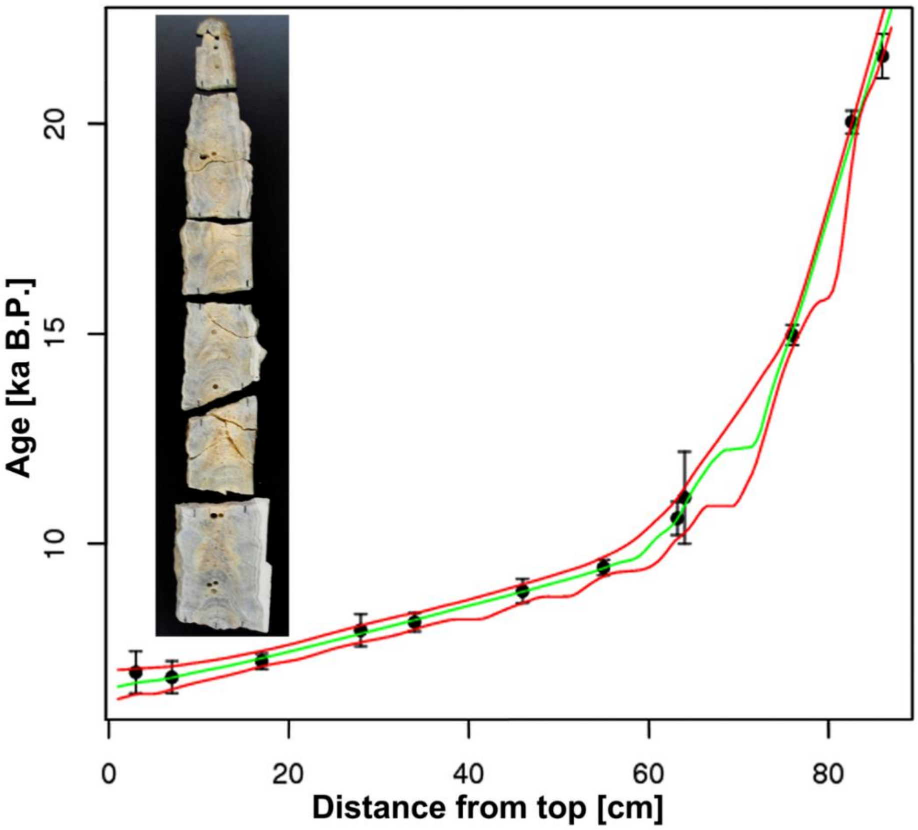

Figure F2 Climate reconstruction in Mawmluh Cave over the 22 to 6 ka period in the stalagmite KM-1 and other relevant caves in the region. (a) “LR04” benthic $\delta^{18}O$ stack (black line[13]) as reference for global climate changes; June insolation at 25° N (dotted red line)[14]; and global atmospheric CO_2 evolution in EPICA Dome C ice (purple line)[15]; (b) records of sea surface temperature using Mg/Ca ratios in the South Bay of Bengal (SBOB; blue line), core SK157-14[16], and West Bay of Bengal (WBOB; green line), core VM29-19[17]; (c) Paleotemperature record using TEX_{86} and MBT/CBT indices and respective calibrations proposed by Blyth and Schouten[8] for cave MAAT (Table S2, equation S4 for TEX_{86} based MAAT, red line; and equation S12 for MBT/CBT based MAAT, brown line) in KM-1 (d) stalagmite growth rate (grey dashed line), $\delta^{13}C$ (brown line) and $\delta^{18}O$ (black line); (e) normalized abundances of iso- (thick grey line) and branched (dashed grey line) GDGTs. Data are from this study unless stated otherwise. See Fig. 1 for cave locations. LGM – Last Glacial Maximum, BA – Bølling-Allerød, YD - Younger Dryas.

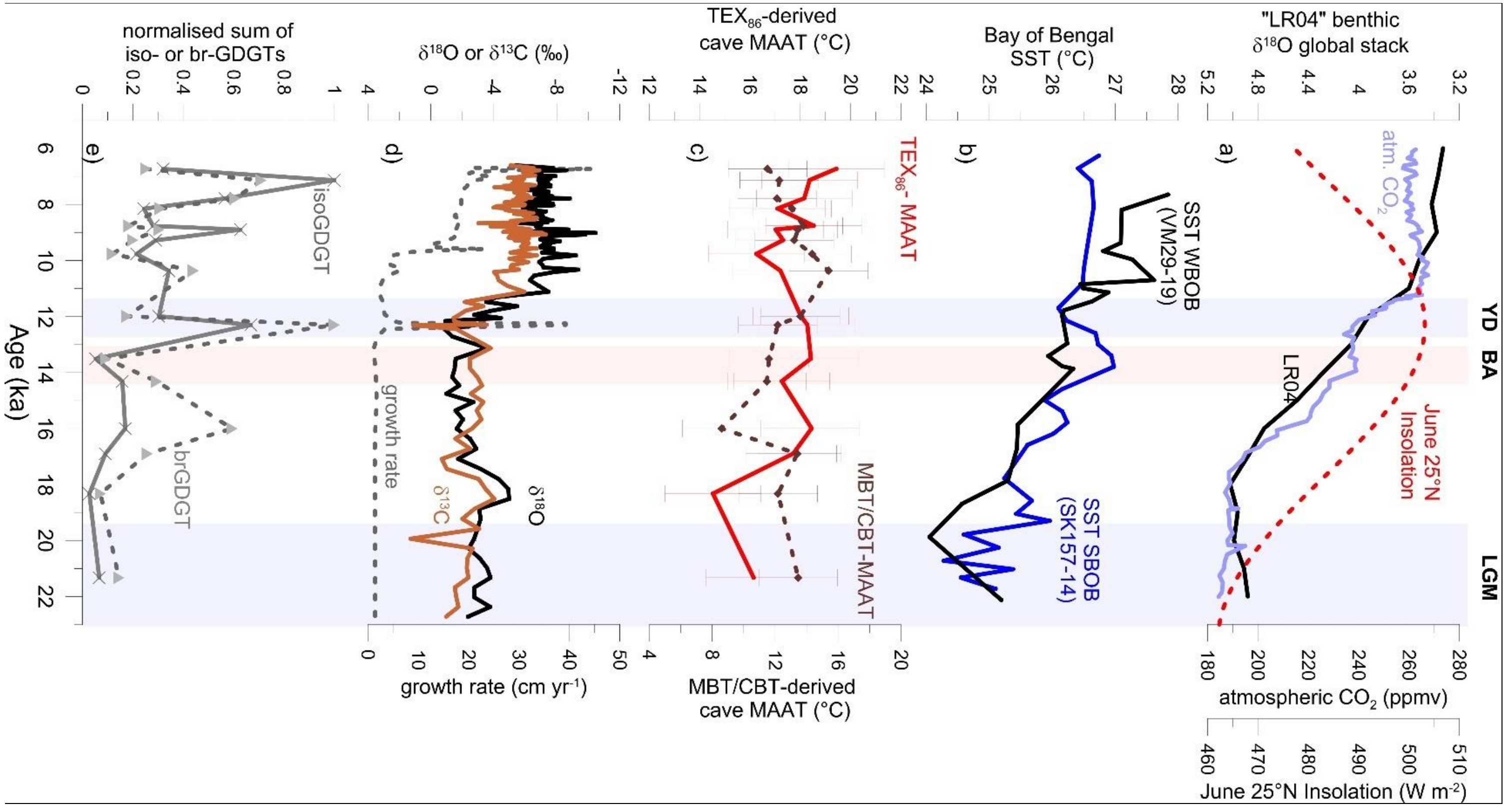

Figure F3 Relationship between GDGT-based paleo-proxies, TEX_{86}, MBT and BIT with carbon and oxygen isotopes during the 22 – 6 ka period investigated in KM-1. Thick crosses indicate samples from the pre-12 ka including the Last Glacial Maximum; light crosses indicate samples from the post-12 ka Holocene period.

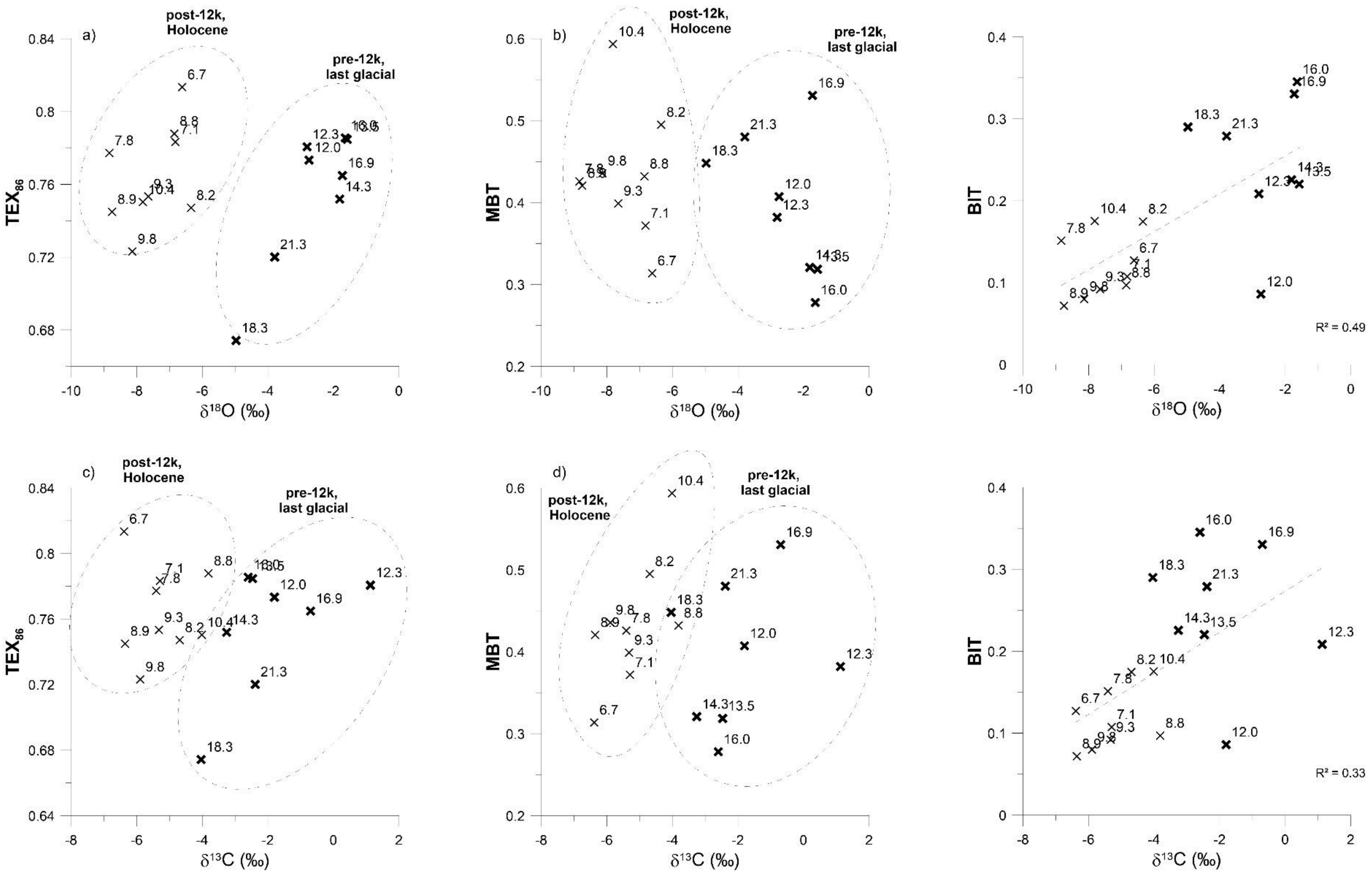

References

1. Scholz, D. & Hoffmann, D. L. StalAge - An algorithm designed for construction of speleothem age models. *Quat. Geochronol.* **6,** 369–382 (2011).
2. Scholz, D. & Hoffmann, D. ^{230}Th/U-dating of fossil corals and speleothems. *Quat. Sci. J.* **57,** 52–76 (2008).
3. Hendy, C. The isotopic geochemistry of speleothems - I. The calculation of the effects of different modes of formaion on the isotopic composition of speleothems and their applicability as paleoclimatic indicators. *Geochim. Cosmochim. Acta* **35,** 801–824 (1971).
4. Rangarajan, R., Ghosh, P. & Naggs, F. Seasonal variability of rainfall recorded in growth bands of the Giant African Land Snail *Lissachatina fulica* (Bowdich) from India. *Chem. Geol.* **357,** 223–230 (2013).
5. Blyth, A. J., Farrimond, P. & Jones, M. An optimised method for the extraction and analysis of lipid biomarkers from stalagmites. *Org. Geochem.* **37,** 882–890 (2006).
6. Hopmans, E. C., Schouten, S., Pancost, R. D., van der Meer, M. T. & Sinninghe Damste, J. S. Analysis of intact tetraether lipids in archaeal cell material and sediments by high performance liquid chromatography/atmospheric pressure chemical ionization mass spectrometry. *Rapid Commun Mass Spectrom* **14,** 585–589 (2000).
7. Schouten, S., Huguet, C., Hopmans, E. C., Kienhuis, M. V. M. & Damste, J. S. S. Analytical methodology for TEX86 paleothermometry by High-Performance Liquid Chromatography / Atmospheric Pressure Chemical Ionization-Mass Spectrometry. *Anal. Chem.* **79,** 2940–2944 (2007).
8. Blyth, A. J. & Schouten, S. Calibrating the glycerol dialkyl glycerol tetraether temperature signal in speleothems. *Geochim. Cosmochim. Acta* **109,** 312–328 (2013).
9. Schouten, S., Hopmans, E. C., Schefuß, E. & Sinninghe Damsté, J. S. Distributional veriations in marine crenarchaeol membrane lipids: a new tool for reconstructing ancient sea water temperatures? *Earth Planet. Sci. Lett.* **204,** 265–274 (2002).
10. Weijers, J. W. H., Schouten, S., van den Donker, J. C., Hopmans, E. C. & Sinninghe Damsté, J. S. Environmental controls on bacterial tetraether membrane lipid distribution in soils. *Geochim. Cosmochim. Acta* **71,** 703–713 (2007).
11. Hopmans, E. C. *et al.* A novel proxy for terrestrial organic matter in sediments based on branched and isoprenoid tetraether lipids. *Earth Planet. Sci. Lett.* **224,** 107–116 (2004).
12. Peterse, F. *et al.* Revised calibration of the MBT-CBT paleotemperature proxy based on branched tetraether membrane lipids in surface soils. *Geochim. Cosmochim. Acta* **96,** 215–229 (2012).
13. Lisiecki, L. E. & Raymo, M. E. A Pliocene-Pleistocene stack of 57 globally distributed benthic ^{18}O records. *Paleoceanography* **20,** 1–17 (2005).
14. Laskar, J. *et al.* A long-term numerical solution for the insolation quantities of the Earth. *Astron. Astrophys.* **428,** 261–285 (2004).
15. Monnin, E. Atmospheric CO_2 concentrations over the Last Glacial Termination. *Science* **291,** 112–114 (2001).
16. Raza, T. *et al.* Glacial to Holocene changes in sea surface temperature and seawater δ^{18}O in the northern Indian Ocean. *Palaeogeogr. Palaeoclimatol. Palaeoecol.* **485,** 697–705 (2017).
17. Rashid, H., England, E., Thompson, L. & Polyak, L. Late glacial to Holocene Indian summer monsoon variability based upon sediment.

Terr. Atmos. Ocean Sci. **22,** 215–228 (2011).
